# 4,4′-[Ethylenebis(nitrilomethylidyne)]dibenzonitrile

**DOI:** 10.1107/S1600536809007284

**Published:** 2009-03-06

**Authors:** Reza Kia, Hoong-Kun Fun, Hadi Kargar

**Affiliations:** a X-ray Crystallography Unit, School of Physics, Universiti Sains Malaysia, 11800 USM, Penang, Malaysia; b Department of Chemistry, School of Science, Payame Noor University (PNU), Ardakan, Yazd, Iran

## Abstract

The mol­ecule of the title Schiff base compound, C_18_H_14_N_4_, lies across a crystallographic inversion centre and adopts an *E* configuration with respect to the azomethine (C=N) bonds. The imino groups are coplanar with the aromatic rings with a maximum deviation of 0.1574 (12) Å for the N atom. Within the mol­ecule, the planar units are parallel, but extend in opposite directions from the dimethyl­ene bridge. In the crystal structure, pairs of inter­molecular C—H⋯N hydrogen bonds link neighbouring mol­ecules into centrosymmetric dimers with *R* ^2^_2_(10) ring motifs. An inter­esting feature of the crystal structure is the short inter­molecular C⋯C inter­action with a distance of 3.3821 (13) Å, which is shorter than the sum of the van der Waals radius of a carbon atom.

## Related literature

For bond-length data, see Allen *et al.* (1987 ▶). For hydrogen-bond motifs, see: Bernstein *et al.* (1995 ▶). For related structures see, for example: Fun & Kia (2008 ▶): Fun, Kargar & Kia (2008 ▶); Fun, Kia & Kargar (2008 ▶). For information on Schiff base complexes and their applications, see, for example, Pal *et al.* (2005 ▶); Calligaris & Randaccio, (1987 ▶). Hou *et al.* (2001 ▶); Ren *et al.* (2002 ▶). For the stability of the temperature controller used for the data collection, see: Cosier & Glazer (1986 ▶).
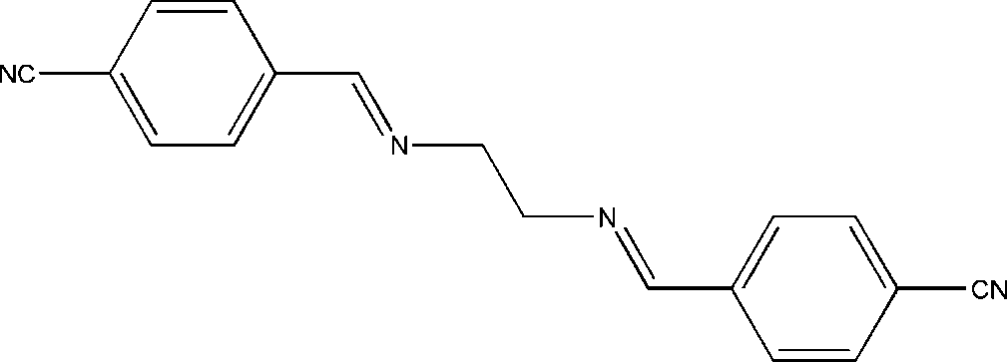


## Experimental

### 

#### Crystal data

C_18_H_14_N_4_
*M* *_r_* = 286.33
Triclinic, 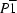

*a* = 4.6843 (2) Å
*b* = 6.9872 (3) Å
*c* = 11.6208 (5) Å
α = 78.147 (3)°
β = 87.462 (3)°
γ = 74.081 (2)°
*V* = 357.94 (3) Å^3^
*Z* = 1
Mo *K*α radiation
μ = 0.08 mm^−1^
*T* = 100 K
0.45 × 0.29 × 0.06 mm

#### Data collection

Bruker SMART APEXII CCD area-detector diffractometer
Absorption correction: multi-scan (*SADABS*; Bruker, 2005 ▶) *T*_min_ = 0.964, *T*_max_ = 0.995
7927 measured reflections
2551 independent reflections
2034 reflections with *I* > 2σ(*I*)
*R*_int_ = 0.023

#### Refinement

*R*[*F* ^2^ > 2σ(*F* ^2^)] = 0.047
*wR*(*F* ^2^) = 0.146
*S* = 1.08
2551 reflections
100 parameters
H-atom parameters constrained
Δρ_max_ = 0.44 e Å^−3^
Δρ_min_ = −0.25 e Å^−3^

### 

Data collection: *APEX2* (Bruker, 2005 ▶); cell refinement: *SAINT* (Bruker, 2005 ▶); data reduction: *SAINT*; program(s) used to solve structure: *SHELXTL* (Sheldrick, 2008 ▶); program(s) used to refine structure: *SHELXTL*; molecular graphics: *SHELXTL*; software used to prepare material for publication: *SHELXTL* and *PLATON* (Spek, 2009).

## Supplementary Material

Crystal structure: contains datablocks global, I. DOI: 10.1107/S1600536809007284/at2733sup1.cif

Structure factors: contains datablocks I. DOI: 10.1107/S1600536809007284/at2733Isup2.hkl

Additional supplementary materials:  crystallographic information; 3D view; checkCIF report

## Figures and Tables

**Table 1:** Hydrogen-bond geometry (Å, °)

*D*—H⋯*A*	*D*—H	H⋯*A*	*D*⋯*A*	*D*—H⋯*A*
C4—H4*A*⋯N2^i^	0.93	2.60	3.4702 (12)	156

Symmetry code: (i) 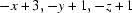.

**Fig. 1.**
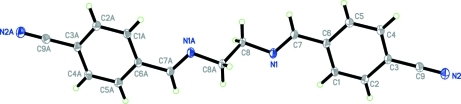
The molecular structure of (I) with atom labels and 50% probability ellipsoids for non-H atoms. The suffix A corresponds to symmetry code [-x + 1, -y, -z].

**Fig. 2.**
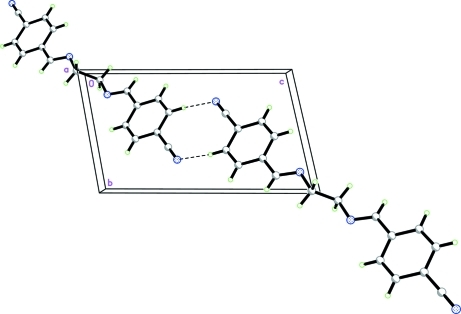
The crystal packing of (I), viewed approximately down the a-axis, showing dimer formation by R22(10) ring motif. Intermolecular interactions are shown as dashed lines.

## supplementary crystallographic information

### Comment

Schiff bases are among the most prevalent mixed-donor ligands in the field of coordination chemistry in which there has been growing interest, mainly because of their wide application in the areas such as biochemistry, synthesis, and catalysis (Pal *et al.*, 2005; Hou *et al.*, 2001; Ren *et al.*, 2002). Many Schiff base complexes have been structurally characterized, but only a relatively small number of free Schiff bases have had their X-ray structures reported (Calligaris & Randaccio, 1987). As an extension of our work (Fun, Kargar & Kia 2008; Fun, Kia & Kargar 2008) on the structural characterization of Schiff base ligands, the title compound (I), is reported here.

The molecule of the title compound, (Fig. 1), lies across a crystallographic inversion centre and adopts an *E* configuration with respect to the azomethine (C═N) bond. The bond lengths (Allen *et al.*, 1987) and angles are within normal ranges and are comparable with the values found in related structures (Fun & Kia 2008; Fun, Kargar & Kia 2008; Fun, Kia & Kargar 2008). The two planar units are parallel but extend in opposite directions from the dimethylene bridge. The interesting feature of the crystal structure is the short intermolecular C3···C6 interactions [symmetry code: 1 + x, y, z] with a distance of 3.3821 (13) Å, which is shorter than the sum of the van der Waals radius of carbon atom. In the crystal structure, pairs of intermolecular C—H···N hydrogen bonds link neighbouring molecules into dimer with *R^2^_2_(10)* ring motif (Bernstein *et al.*, 1995) (Table 1, Fig. 2).

### Experimental

The synthetic method has been described earlier (Fun, Kargar, & Kia, 2008). Single crystals suitable for *X*-ray diffraction were obtained by evaporation of an ethanol solution at room temperature.

### Refinement

All of the hydrogen atoms were positioned geometrically with C—H = 0.95 or 0.97 Å and refined in riding mode with *U*_iso_ (H) = 1.2 *U*_eq_ (C).

### Crystal data

**Table:**

C_18_H_14_N_4_	*Z* = 1
*M**_r_* = 286.33	*F*(000) = 150
Triclinic, *P*1	*D*_x_ = 1.328 Mg m^−^^3^
Hall symbol: -P 1	Mo *K*α radiation, λ = 0.71073 Å
*a* = 4.6843 (2) Å	Cell parameters from 3276 reflections
*b* = 6.9872 (3) Å	θ = 3.1–36.3°
*c* = 11.6208 (5) Å	µ = 0.08 mm^−^^1^
α = 78.147 (3)°	*T* = 100 K
β = 87.462 (3)°	Plate, colourless
γ = 74.081 (2)°	0.45 × 0.29 × 0.06 mm
*V* = 357.94 (3) Å^3^	

### Data collection

**Table:**

Bruker SMART APEXII CCD area-detector diffractometer	2551 independent reflections
Radiation source: fine-focus sealed tube	2034 reflections with *I* > 2σ(*I*)
graphite	*R*_int_ = 0.023
φ and ω scans	θ_max_ = 32.5°, θ_min_ = 3.1°
Absorption correction: multi-scan (*SADABS*; Bruker, 2005)	*h* = −6→7
*T*_min_ = 0.964, *T*_max_ = 0.995	*k* = −10→10
7927 measured reflections	*l* = −17→17

### Refinement

**Table:**

Refinement on *F*^2^	Primary atom site location: structure-invariant direct methods
Least-squares matrix: full	Secondary atom site location: difference Fourier map
*R*[*F*^2^ > 2σ(*F*^2^)] = 0.047	Hydrogen site location: inferred from neighbouring sites
*wR*(*F*^2^) = 0.146	H-atom parameters constrained
*S* = 1.08	*w* = 1/[σ^2^(*F*_o_^2^) + (0.0885*P*)^2^ + 0.0252*P*] where *P* = (*F*_o_^2^ + 2*F*_c_^2^)/3
2551 reflections	(Δ/σ)_max_ < 0.001
100 parameters	Δρ_max_ = 0.44 e Å^−^^3^
0 restraints	Δρ_min_ = −0.25 e Å^−^^3^

### Special details

**Table:**

Experimental. The crystal was placed in the cold stream of an Oxford Cyrosystems Cobra open-flow nitrogen cryostat (Cosier & Glazer, 1986) operating at 100.0 (1) K.
Geometry. All esds (except the esd in the dihedral angle between two l.s. planes) are estimated using the full covariance matrix. The cell esds are taken into account individually in the estimation of esds in distances, angles and torsion angles; correlations between esds in cell parameters are only used when they are defined by crystal symmetry. An approximate (isotropic) treatment of cell esds is used for estimating esds involving l.s. planes.
Refinement. Refinement of F^2^ against ALL reflections. The weighted R-factor wR and goodness of fit S are based on F^2^, conventional R-factors R are based on F, with F set to zero for negative F^2^. The threshold expression of F^2^ > 2sigma(F^2^) is used only for calculating R-factors(gt) etc. and is not relevant to the choice of reflections for refinement. R-factors based on F^2^ are statistically about twice as large as those based on F, and R- factors based on ALL data will be even larger.

### Fractional atomic coordinates and isotropic or equivalent isotropic displacement parameters (Å^2^)

**Table:**

	*x*	*y*	*z*	*U*_iso_*/*U*_eq_	
N1	0.59056 (17)	0.17735 (11)	0.08275 (6)	0.01615 (18)	
N2	1.42099 (19)	0.74379 (12)	0.37951 (7)	0.0216 (2)	
C1	0.8714 (2)	0.45474 (13)	0.15338 (7)	0.01522 (19)	
H1A	0.7912	0.4927	0.0776	0.018*	
C2	1.0275 (2)	0.57187 (13)	0.19178 (8)	0.01621 (19)	
H2A	1.0552	0.6873	0.1416	0.019*	
C3	1.14366 (19)	0.51580 (13)	0.30666 (7)	0.01489 (19)	
C4	1.1063 (2)	0.34172 (13)	0.38257 (7)	0.01654 (19)	
H4A	1.1845	0.3047	0.4586	0.020*	
C5	0.9504 (2)	0.22467 (13)	0.34268 (8)	0.01632 (19)	
H5A	0.9235	0.1088	0.3927	0.020*	
C6	0.83369 (19)	0.27878 (13)	0.22843 (7)	0.01374 (18)	
C7	0.67371 (19)	0.14854 (13)	0.18937 (7)	0.01451 (18)	
H7A	0.6322	0.0424	0.2440	0.017*	
C8	0.4324 (2)	0.03967 (13)	0.05429 (7)	0.01563 (19)	
H8A	0.2243	0.1108	0.0394	0.019*	
H8B	0.4460	−0.0732	0.1202	0.019*	
C9	1.2999 (2)	0.64058 (13)	0.34745 (7)	0.01641 (19)	

### Atomic displacement parameters (Å^2^)

**Table:**

	*U*^11^	*U*^22^	*U*^33^	*U*^12^	*U*^13^	*U*^23^
N1	0.0164 (4)	0.0181 (3)	0.0170 (3)	−0.0075 (3)	−0.0010 (3)	−0.0063 (3)
N2	0.0229 (4)	0.0232 (4)	0.0216 (4)	−0.0100 (3)	−0.0030 (3)	−0.0054 (3)
C1	0.0149 (4)	0.0185 (4)	0.0139 (4)	−0.0058 (3)	−0.0021 (3)	−0.0047 (3)
C2	0.0167 (4)	0.0168 (4)	0.0169 (4)	−0.0068 (3)	−0.0011 (3)	−0.0040 (3)
C3	0.0127 (4)	0.0173 (4)	0.0168 (4)	−0.0051 (3)	−0.0004 (3)	−0.0067 (3)
C4	0.0169 (4)	0.0191 (4)	0.0151 (4)	−0.0059 (3)	−0.0028 (3)	−0.0047 (3)
C5	0.0175 (4)	0.0166 (4)	0.0159 (4)	−0.0063 (3)	−0.0015 (3)	−0.0029 (3)
C6	0.0120 (4)	0.0157 (4)	0.0149 (4)	−0.0040 (3)	−0.0001 (3)	−0.0056 (3)
C7	0.0142 (4)	0.0159 (4)	0.0153 (4)	−0.0059 (3)	0.0003 (3)	−0.0050 (3)
C8	0.0159 (4)	0.0183 (4)	0.0162 (4)	−0.0085 (3)	−0.0008 (3)	−0.0058 (3)
C9	0.0158 (4)	0.0182 (4)	0.0159 (4)	−0.0047 (3)	−0.0012 (3)	−0.0046 (3)

### Geometric parameters (Å, °)

**Table:**

N1—C7	1.2745 (11)	C4—C5	1.3893 (12)
N1—C8	1.4585 (11)	C4—H4A	0.9300
N2—C9	1.1551 (11)	C5—C6	1.3962 (12)
C1—C2	1.3821 (11)	C5—H5A	0.9300
C1—C6	1.4031 (12)	C6—C7	1.4730 (11)
C1—H1A	0.9300	C7—H7A	0.9300
C2—C3	1.4017 (12)	C8—C8^i^	1.5246 (16)
C2—H2A	0.9300	C8—H8A	0.9700
C3—C4	1.3966 (12)	C8—H8B	0.9700
C3—C9	1.4389 (11)		
			
C7—N1—C8	117.00 (7)	C6—C5—H5A	119.6
C2—C1—C6	120.14 (8)	C5—C6—C1	119.61 (8)
C2—C1—H1A	119.9	C5—C6—C7	118.94 (7)
C6—C1—H1A	119.9	C1—C6—C7	121.45 (8)
C1—C2—C3	119.68 (8)	N1—C7—C6	121.78 (8)
C1—C2—H2A	120.2	N1—C7—H7A	119.1
C3—C2—H2A	120.2	C6—C7—H7A	119.1
C4—C3—C2	120.80 (8)	N1—C8—C8^i^	109.60 (9)
C4—C3—C9	119.58 (8)	N1—C8—H8A	109.8
C2—C3—C9	119.61 (7)	C8^i^—C8—H8A	109.8
C5—C4—C3	118.95 (8)	N1—C8—H8B	109.8
C5—C4—H4A	120.5	C8^i^—C8—H8B	109.8
C3—C4—H4A	120.5	H8A—C8—H8B	108.2
C4—C5—C6	120.81 (8)	N2—C9—C3	178.76 (9)
C4—C5—H5A	119.6		
			
C6—C1—C2—C3	−1.03 (13)	C4—C5—C6—C7	179.13 (7)
C1—C2—C3—C4	0.65 (13)	C2—C1—C6—C5	1.07 (13)
C1—C2—C3—C9	−178.50 (7)	C2—C1—C6—C7	−178.76 (7)
C2—C3—C4—C5	−0.28 (13)	C8—N1—C7—C6	−179.67 (7)
C9—C3—C4—C5	178.86 (7)	C5—C6—C7—N1	−172.24 (8)
C3—C4—C5—C6	0.32 (14)	C1—C6—C7—N1	7.59 (14)
C4—C5—C6—C1	−0.71 (14)	C7—N1—C8—C8^i^	−131.42 (10)

Symmetry codes: (i) −*x*+1, −*y*, −*z*.

### Hydrogen-bond geometry (Å, °)

**Table:**

*D*—H···*A*	*D*—H	H···*A*	*D*···*A*	*D*—H···*A*
C4—H4A···N2^ii^	0.93	2.60	3.4702 (12)	156

Symmetry codes: (ii) −*x*+3, −*y*+1, −*z*+1.
